# Hijacking Extracellular Targeted Protein Degrader–Drug Conjugates for Enhanced Drug Delivery

**DOI:** 10.1021/jacs.5c15047

**Published:** 2025-10-15

**Authors:** Fangzhu Zhao, Yan Wu, Kaitlin Schaefer, Yun Zhang, Kun Miao, Zi Yao, Snehal D. Ganjave, Kaan Kumru, Trenton M. Peters-Clarke, Alex Inague, James A. Olzmann, Kevin K. Leung, James A. Wells

**Affiliations:** † Department of Pharmaceutical Chemistry, 8785 University of California San Francisco, San Francisco, California 94158, United States; ‡ Department of Molecular and Cell Biology, 1438 University of California Berkeley, Berkeley, California 94720, United States; § Department of Nutritional Sciences and Toxicology, 1438 University of California Berkeley, Berkeley, California 94720, United States; ∥ Department of Cellular & Molecular Pharmacology, 8785 University of California San Francisco, San Francisco, California 94158, United States

## Abstract

Antibody-based therapeutics encompass diverse modalities for targeting tumor cells. Among these, antibody–drug conjugates (ADCs) and extracellular targeted protein degradation (eTPD) specifically depend on efficient lysosomal trafficking for activity. A major limitation of ADCs is their reliance on antigens with efficient internalization, while eTPD approaches, although capable of trafficking diverse targets to lysosomes, lack cytotoxic potency. To address this, we developed degrader–drug conjugates (DDCs), leveraging the endocytic and recycling activities of eTPD to enhance lysosomal delivery. We utilized fast internalizers, the low-density lipoprotein receptor (LDLR) and the chemokine receptor (CXCR7), to enhance lysosomal delivery. LDLR-based degraders enabled efficient and selective degradation of diverse extracellular membrane proteins, while DDCs with cytotoxic payload enhanced cytotoxicity compared to conventional ADCs *in vitro*. This dual modality addresses key challenges of inadequate internalization in conventional ADCs and cytotoxic potency in current eTPD strategies. Our findings demonstrate that DDCs provide additional optionality for developing next-generation antibody therapeutics with broader utility and improved efficacy in cancer treatment.

## Graphical abstract

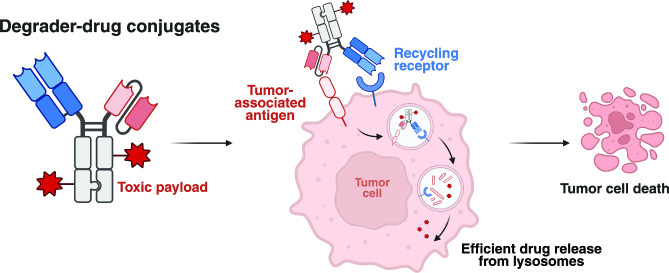

## Introduction

Extracellular and membrane-associated proteins represent approximately one-third of all protein-coding genes and are key targets for antibody-based therapeutics.^1^ Antibodies provide diverse mechanisms of action in cancer therapy, including receptor inhibition or activation,^2^ immune cell recruitment,^3^ antibody–drug conjugates (ADCs) for targeted toxin delivery,^4^ and extracellular targeted protein degradation (eTPD) for proteolytic degradation.^5^ Among these, ADCs combine tumor-targeting antibodies with cytotoxic drugs to achieve selective tumor cell killing.^6^ However, the efficacy of ADCs hinges on the efficient internalization of the antibody–antigen complex to facilitate intracellular drug release in the lysosome. Not all surface antigens undergo productive internalization upon antibody binding, posing a significant limitation to ADC effectiveness.^7^ Recent innovations in bispecific antibodies have sought to overcome this hurdle by enhancing receptor internalization through receptor clustering, such as biparatopic ADCs,^8,9^ or by targeting fast-internalizing receptors in dual-antigen strategies for more efficient lysosomal trafficking.^10,11^

In parallel, eTPD has emerged as a promising therapeutic approach that co-opts natural endolysosomal pathways to selectively degrade membrane-bound and soluble extracellular proteins. eTPD utilizes a wide array of cell surface degrader systems including: neonatal Fc receptor (FcRn),^12^ glycan binding receptors,^13–15^ transmembrane E3 ligases,^16,17^ cytokine receptors,^18^ integrins,^19^ and transferrin receptors.^20^ Increasing the optionality of cell surface degraders offers a greater opportunity for cell-specific targeting. However, unlike ADCs or bispecific T cell engagers that deliver cytotoxic payloads or recruit immune effectors, eTPD efficacy depends on the biological importance of the target itself. Effective targets are typically those essential for tumor growth or survival, such as mutant epidermal growth factor receptor (EGFR) with constitutive autophosphorylation^20^ or human epidermal growth factor receptor 2 (HER2)^18^ amplification. This requirement substantially narrows the pool of viable targets for therapeutic eTPD, which also exhibits markedly weaker potency compared to that of ADCs.

Given that ADCs with cleavable linkers rely on efficient lysosomal trafficking and that eTPD excels at directing targets into this pathway, we sought to harness the efficient lysosomal delivery capacity of eTPD to improve ADC payload delivery. To this end, we developed degrader–drug conjugates (DDCs), a new class of bifunctional therapeutics that intentionally hybridize eTPD with ADC for greater efficiency of drug payload delivery.

As a proof-of-concept, we exploited the robust recycling low-density lipoprotein receptor (LDLR) as a lysosomal trafficking effector. LDLR naturally internalizes LDL via clathrin-mediated endocytosis^21,22^ and facilitates its delivery to the lysosome at low pH.^23^ LDLR is upregulated in proliferating cancer cells^24,25^ and activated T cells.^26^ It is one of the fastest and most efficient internalizers that recycles through the lysosome every 12 min.^27^ These features make the LDLR an attractive candidate for DDCs, which we refer to as LDLR-targeting chimeras (LIPTACs). We show that LIPTACs mediate selective and efficient lysosomal degradation for multiple membrane proteins. Moreover, by conjugating cytotoxic payloads to either LIPTACs or cytokine receptor-targeting chimeras (KineTACs),^18^ we show that DDCs can boost the potencies of conventional ADCs by up to 20-fold *in vitro*. Together, our findings highlight DDCs as a hybrid modality that may represent a new path forward to both enhance payload delivery and broaden the therapeutic utility of antibody-based modalities.

## Results

### Selection and Characterization of LDLR Antibodies

The extracellular portion of the LDLR contains the ligand binding domain, the epidermal growth factor (EGF)-like domain, and the O-linked sugar domain.^28^ We and others have observed that the ligand-binding domain of the LDLR can be shed to varying degrees^29,30^ in cells transformed with *KRAS­(G12V)* or *HER2*. This prompted us to select for antibodies against the membrane proximal EGF-like domain of the LDLR in order to preserve its ligand ability for LDL uptake and to enable recruitment of both full-length and cleaved forms (cLDLR) for eTPD. After four rounds of phage selection, we plated 96 single phage colonies for screening using an enzyme-linked immunosorbent assay (ELISA) (Figure S1a,b).

Phage that passed initial screening were expressed recombinantly as monoclonal fragment antigen-binding (Fab) antibodies for further characterization (Table S1). The ELISA binding assay against the cLDLR or full-length (flLDLR) identified multiple high-affinity binding-affinity clones (Figure 1a, Figure S2a). Flow cytometry confirmed that all Fabs bound to the MDA-MB-231 cells (Figure 1b). None of the Fabs exhibited cross-reactivity with the other members of the LDLR family, including the LDLR-related protein 2 (LRP2), LRP8, and VLDLR (Figure S2a). All the Fabs displayed minimal polyreactive binding across the nonspecific antigen panels^31^ (Figure S2b). Epitope binning revealed that 142F6 recognized a distinct epitope, whereas all other clones bound the same LDLR epitope (Figure S3). Among them, 142F1 showed the highest affinity by ELISA, and together with 142F6 was prioritized for further characterization. Notably, 142F1 and 142F6 bound simultaneously, consistent with recognition of two distinct, noncompeting epitopes (Figure 1c). Bio-layer interferometry (BLI) experiments showed that 142F1 and 142F6 bound to cLDLR with dissociation constants of 5.8 and 16 nM, respectively (Figure 1d).

**Figure 1:**
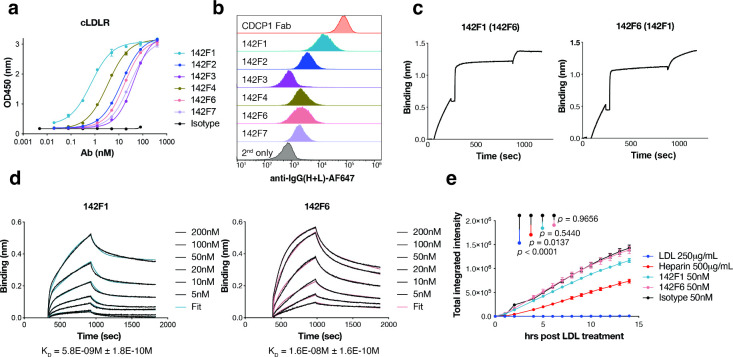
Characterization of LDLR-specific antibodies. (a) ELISA binding of recombinant Fabs against the cLDLR antigen. Absorbance was read at 450 nm. An anti-CDCP1 Fab 4A06 ^52^ was used as a negative isotype control. Each sample was tested in biological duplicate and error bars represented standard deviations. (b) Flow cytometry of different Fabs binding to LDLR^+^ MDA-MB-231 cells. Then 50 nM of each Fab was incubated with cells for 30 min and washed twice, followed by AF647 conjugated goat antihuman IgG (H+L) antibody staining for 15 min. (c) Epitope binning of two anti-LDLR Fabs, 142F1 and 142F6, revealed two different epitopes on cLDLR. Biotinylated cLDLR was captured using a streptavidin biosensor and indicated antibodies at a concentration of 200 nM were incubated for 10 min followed by incubation with 50 nM of the second competing antibodies for 5 min. (d) BLI analysis of 142F1 and 142F6 Fabs to estimate their affinities to cLDLR. Biontinylated cLDLR was immobilized via the streptavidin biosensor and varying concentrations of each Fab was injected. Black lines were the experimental trace obtained from the BLI experiments and colored lines were the global fits. (e) Internalization of pH-sensitive Phrodo red-labeled LDL on HeLa cells after 30 min pretreatment with LDL, heparin, or each Fab, respectively. Total integrated intensity is calculated by ROCU × μm^2^/image on the Incucyte software. Each sample was tested in biological triplicate and error bars represent standard deviations. Statistics were calculated by one-way ANOVA and Holm–Sidak multiple comparisons test.

Given that LDLR is essential for regulating plasma cholesterol levels, we then investigated whether our LDLR Fabs affected LDL uptake. We used an Incuyte-based uptake assay with fluorescently labeled (pHrodo) LDL to monitor trafficking to low pH vesicles. We serum starved Hela cells, treated them with LDLR Fabs, heparin that binds LDL and blocks LDLR binding,^32,33^ or unlabeled competitor LDL respectively, and then added pHrodo red dye-labeled LDL. As expected, pHrodo red dye-labeled LDL trafficked robustly in both the presence and absence of LDLR Fabs, but was inhibited upon addition of unlabeled LDL and heparin (Figure 1e). These data suggest that the LDLR-mediated LDL trafficking remains intact upon Fab binding.

### Design of LIPTAC Degraders for EGFR Degradation

The design of LIPTACs involved a bispecific antibody, with one arm targeting a protein of interest (POI) and the other arm recruiting LDLR to bring the POI and LDLR in close proximity for lysosomal trafficking and eTPD. As a proof-of-concept, we generated LIPTACs to degrade EGFR, a receptor tyrosine kinase that plays a critical role in the development and progression of various types of cancers.^34,35^ The therapeutic anti-EGFR Cetuximab (Ctx) and anti-LDLR antibody (142F1 or 142F6) were fused to heterodimeric Fc domains respectively,^36^ with T350V/L351Y/F405A/Y407V mutations in chain A and T350V/T366L/K392L/T394W mutations in chain B. To eliminate Fc-effector function for macrophage and NK cell recruitment, we introduced the L234A/L235A/P329G mutations (LALAPG)^37^ in both Fc chains. To avoid heavy and light chain mispairing, one arm was designed as a single chain variable fragment (scFv) and the other in Fab format (Figure 2a). We produced four Ctx-LIPTAC formats, as shown in Figure S4a and evaluated their degradation efficiency in HeLa cells. After 24 h treatment with 50 nM of each LIPTAC, levels of EGFR were quantified by Western blotting. We found that LIPTAC1 with anti-LDLR 142F1 as Fab and anti-EGFR Ctx in scFv exhibited the greatest EGFR degradation (Figure S4b). This improvement may result from both the higher binding affinity and distinct epitope specificity^5,18^ of the LDLR antibody. A dose–response curve for LIPTAC1 at 24h revealed that LIPTAC1 retained potent EGFR degradation activity at concentrations as low as 5 nM (Figure 2b).

**Figure 2:**
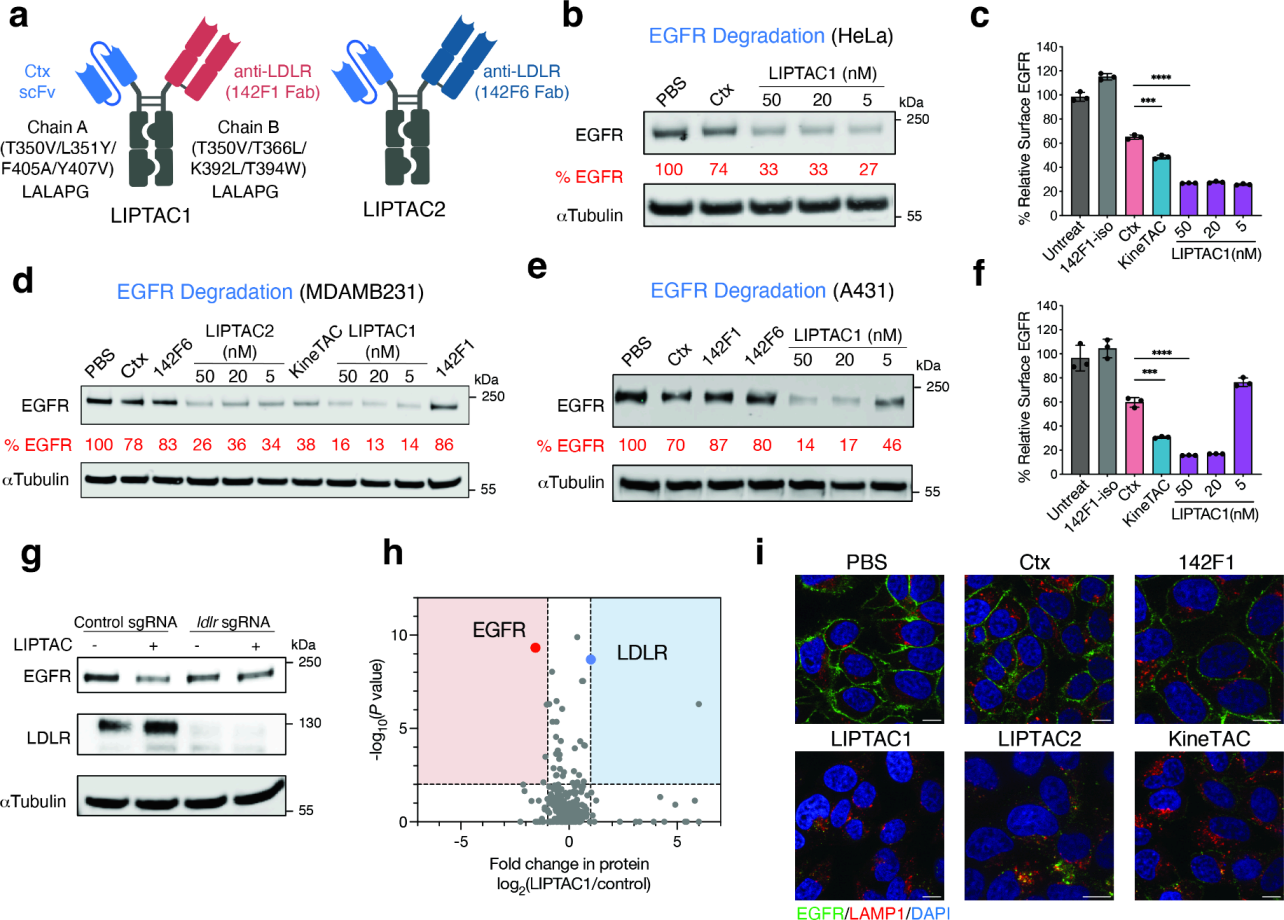
Generation of LIPTACs for degradation of EGFR. (a) Schematic illustration of LDLR-Ctx LIPTAC bispecific constructs. (b) Western blot showing degradation of total EGFR on HeLa cells following 24 h of treatment with Ctx-LIPTAC or 50 nM control antibodies. Data represents three biological replicates. Percent EGFR levels were quantified by ImageJ relative to PBS control. (c) Changes in surface EGFR based on flow cytometry analysis on MDA-MB-231 cells following 24 h of 50 nM 142F1 isotype IgG, Ctx IgG, Ctx-LIPTAC, or Ctx-KineTAC treatment. Percent EGFR was determined by median fluorescence intensity (MFI) of the PE fluorescence channel of live cells. Each sample was tested in biological triplicate and error bars represented standard deviation. Statistics were calculated by unpaired two-tailed student *t* test. ***P < 0.001. ****P < 0.0001. (d) Western blot showing degradation of total EGFR on MDA-MB-231 cells after 24 h treatment of 50 nM Ctx, KineTAC, monomeric LDLR isotype, or varying concetrations of LIPTACs. Data represented three biological replicates. (e) EGFR degradation in A431 cells following 24 h of Ctx-LIPTAC1 treatment. Data represents three biological replicates. (f) Flow cytometry analysis showing degradation of surface EGFR on A431 cells following 24 h of Ctx-LIPTACs, 50 nM Ctx IgG, and 50 nM Ctx-KineTAC treatment. Each sample was tested in biological triplicate and error bars represent standard deviations. Statistics were calculated by unpaired two-tailed student *t* test. ***P < 0.001. ****P < 0.0001. (g) Western blot analysis of EGFR and LDLR in LDLR knockout and control HCC1143 cells after 24 h of 5 nM LIPTAC treatment. Data represent two biological replicates. (h) Fold-change in surface protein abundance in MDA-MB-231 cells following 48 h of treatment with or without 50 nM Ctx-LIPTAC1, as measured by quantitative proteomics analysis. N-Linked cell surface glycoproteins were captured by the cell-surface capture technology^39^ and enriched by biocytin hydrazide. Surface proteins were annotated using the SURFY database.^73^ (i) Confocal microscopy images of HeLa cells treated with 50 nM of indicated bispecific antibodies or isotype controls for 24 h. Scale bar, 10 μm.

Given the tumor-associated nature of LDLR, we further tested degradation in multiple cancer cells. LIPTACs exhibited efficient EGFR degradation on triple-negative breast cancer cell line HCC1143 (Figure S4c), the pancreatic cancer cell line PANC-1 (Figure S4d), as well as the nonsmall cell lung cancer cell line NCI-H1975 (Figure S4e).

The Ctx-KineTAC utilizing the CXCL12 cytokine efficiently degrades EGFR using the CXCR7 recycling receptor.^18^ Thus, we compared KineTAC and LIPTAC1. Flow cytometry demonstrated that both LIPTAC1 and the KineTAC efficiently degraded surface EGFR on MDA-MB-231 cells (Figure 2c). LIPTAC1 showed somewhat higher degradation efficiency than KineTAC (Figure 2c), possibly due to LDLR expression being higher than that of CXCR7 on MDA-MB-231 cells. Moreover, LDLR levels did not change, indicating that the LDLR was not consumed in the process (Figure S4f). Similar findings were observed by Western blotting (Figure S4g), suggesting that LDLR was recycled back. Treatment with either arm of the LIPTACs individually at 50 nM did not affect EGFR levels, indicating that both targets must be brought together to cause degradation (Figure 2d). Additionally, LIPTAC1 efficiently degraded EGFR with a maximal percent degradation (*D*_max_) of 86%, on the EGFR high expressing epidermoid carcinoma cell line A431 (Figure 2d,f). To assess the specificity of LDLR-mediated protein degradation, we treated LDLR knockout^38^ (KO) and control HCC1143 cells with LIPTAC1. EGFR degradation was less efficient in LDLR KO cells compared with control Cas 9 cells (Figure 2g), indicating the requirement of LDLR for degradation.

To further investigate the impact of LIPTAC treatment on the proteome, we conducted quantitative mass spectrometry analysis of surface-enriched lysates^39^ following LIPTAC1 treatment in MDA-MB-231 cells. We found that only a few surface proteins showed changes in abundance (Figure S5), with EGFR exhibiting the most significant reduction, supporting the high selectivity of LIPTAC1 (Figure 2h). Interestingly, LDLR levels increased approximately 2-fold (Figure 2h), potentially due to partial competition between the 142F1 antibody and the proprotein convertase subtilisin/kexin type 9 (PCSK9) binding (Figure S6), which may inhibit PCSK9-mediated LDLR degradation.^40,41^ The abundances of other LDLR family members observed in our data set, including LRP1, LRP6, and LRP8, remained unchanged. This data further support the selectivity of the LDLR antibodies.

Immunofluorescence microscopy revealed virtually complete removal of EGFR from the cell surface following 24 h of LIPTAC treatment compared to treatment with PBS, Ctx, or the 142F1 clone, further highlighting that LIPTACs induce robust internalization of target proteins (Figure 2i). The colocalization of lysosomal LAMP1 with intracellular EGFR further supported lysosomal shuttling by LIPTACs. Together, our findings demonstrate that LIPTAC-mediated targeted protein degradation is efficient, selective, and dependent on the LDLR.

### LIPTAC-Mediated Degradation of Multiple Membrane Proteins

We sought to determine whether LIPTAC could degrade other therapeutically relevant cell surface proteins. First, we targeted PD-L1, an immune checkpoint expressed in tumor microenvironment that suppresses cytotoxic T cell function.^42^ We generated a PD-L1-targeting LIPTAC by incorporating anti-PD-L1 Atezolizumab^43^ (Atz) Fab with 142F1 scFv. Atz-LIPTAC efficiently degraded PD-L1 in MDA-MB-231 cells after 24 h of treatment (Figure 3a,b). Additionally, to determine degradation mechanisms, cells were pretreated with Bafilomycin A1 (an inhibitor of lysosome acidification^44^) or MG132 (a proteasome inhibitor^45^) prior to LIPTAC1 treatment. Bafilomycin A1 inhibited PD-L1 degradation, while MG132 did not (Figure 3c), suggesting that LIPTAC-mediated protein degradation occurs predominantly by delivery to the lysosome.

**Figure 3:**
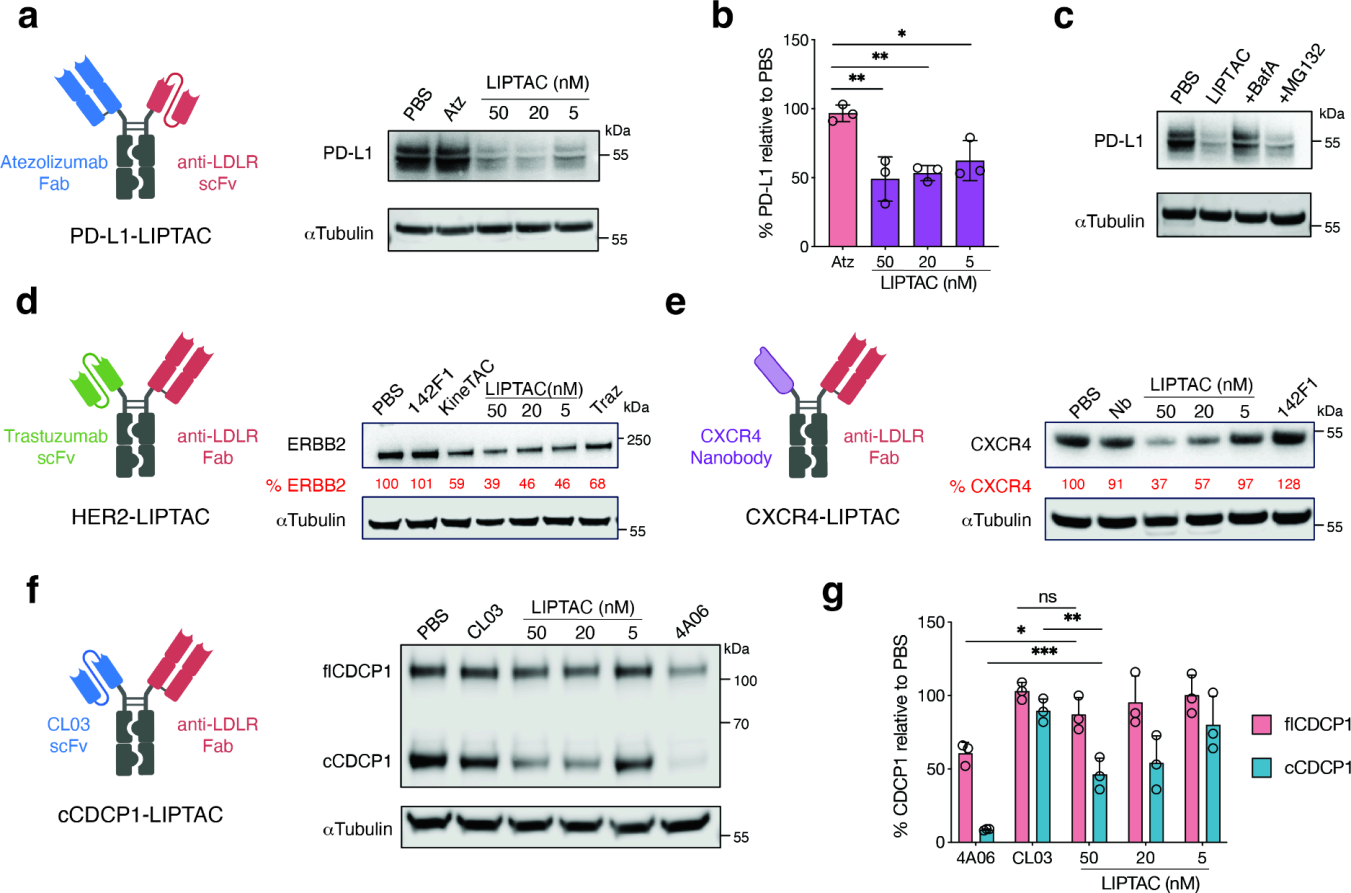
LIPTAC-mediated degradation of multiple extracellular proteins. (a,b) Schematic illustration of PD-L1 targeting LIPTAC, and Western blot showing total degradation of PD-L1 on MDA-MB-231 cells following 24 h treatment of 50 nM monomeric anti-PD-L1 parent, Atz, or LIPTAC containing Atz. Percent PD-L1 levels were quantified by ImageJ relative to PBS control. Each sample was tested in biological triplicate and error bars represent standard deviations. Statistics were calculated by unpaired two-tailed student *t* test. *P < 0.05. **P < 0.01. (c) Western blot analysis showing lysosome-dependent PD-L1 degradation on MDA-MB-231 cells. Cells were pretreated with either 500 nM Bafilomycin A (BafA) or 500 nM MG132 for 1 h followed by 24 h treatment with 50 nM LIPTAC. (d) Schematic illustration of HER2-targeting LIPTAC and Western blot analysis showing total HER2 degradation on MCF7 cells following 24 h treatment of monomeric Traz, LIPTAC and KineTAC. Percent ERBB2 (HER2) levels were quantified by ImageJ relative to PBS control. Data represent at least two independent experiments. (e) Schematic illustration of CXCR4-targeting LIPTAC and Western blot analysis showing total CXCR4 degradation on HeLa cells following 24 h treatment of Nb monomer, monomeric 142F1, or LIPTAC. (f,g) Schematic illustration of cleaved CDCP1 (cCDCP1)-targeting LIPTAC and degradation of CDCP1 in PL45 cells following 24 h treatment of 50 nM cCDCP1 binder CL03 IgG, full-length CDCP1 (flCDCP1) binder 4A06 IgG, or cCDCP1-specific LIPTAC. Each sample was tested in biological triplicate and error bars represent standard deviations. Statistics were calculated by unpaired two-tailed student *t* test. *P < 0.05. **P < 0.01. ns, not significant.

Next, we targeted HER2, which is frequently upregulated in cancer and linked to breast cancer invasiveness and tumor progression.^46^ We generated an HER2-targeting LIPTAC by incorporating anti-HER2 trastuzumab (Traz) scFv with our LDLR Fab. Treatment of MCF7 cells with the Traz-LIPTAC resulted in efficient HER2 degradation (Figure 3d). We then sought to evaluate degradation of multipass transmembrane proteins, such as G protein-coupled receptors (GPCRs). We targeted CXCR4, a chemokine receptor involved in tumor growth and metastasis.^47^ The CXCR4-targeting LIPTAC was built using a CXCR4 antagonizing nanobody (Nb)^48^ on one arm and the LDLR 142F1 Fab on the other arm of the Fc. Western blotting showed significant CXCR4 degradation after 24 h treatment of Nb-LIPTAC, and not by the Nb monomer (Figure 3e). The antigen-targeting arm of LIPTAC can be flexibly incorporated into various antibody formats, including Fab fragments, scFvs, and Nbs.

Additionally, we investigated CUB domain-containing protein 1 (CDCP1), which is highly overexpressed in RAS-driven cancers and undergoes ectodomain cleavage by extracellular proteases on cancer cells but not healthy cells.^49–51^ We previously developed two antibody clones: 4A06, which targets both full-length and cleaved forms of CDCP1, and CL03, which selectively recognizes the cleaved form for enhanced tumor specificity.^52^ We found that 4A06 IgG was efficiently internalized, leading to the downregulation of both full-length and cleaved CDCP1 (Figure 3f). In contrast, CL03 IgG alone did not induce CDCP1 degradation. However, the CL03-based LIPTAC selectively and efficiently degraded cleaved CDCP1, without affecting the full-length form, in PL45 cells (Figure 3 **f,g**). Overall, these results highlight the versatility of the LIPTAC platform in selectively degrading a range of extracellular membrane proteins and even isoforms.

### Improved Modality of Target Cell Killing via Degrader Drug Conjugates

Both toxin delivery by ADCs and protein degradation by eTPD require internalization, delivery, and proteolysis of the lysosome. We hypothesized that eTPD coupled to an ADC could be used to enhance the potency of an ADC in situations where the target of the ADC was not optimized for lysosomal trafficking (Figure 4a). Our data here show that the Ctx-IgG only partially induces EGFR degradation (Figure 2e). Given that Ctx-LIPTAC efficiently degrades EGFR, we selected Ctx ADC as a test case and a DDC.

**Figure 4:**
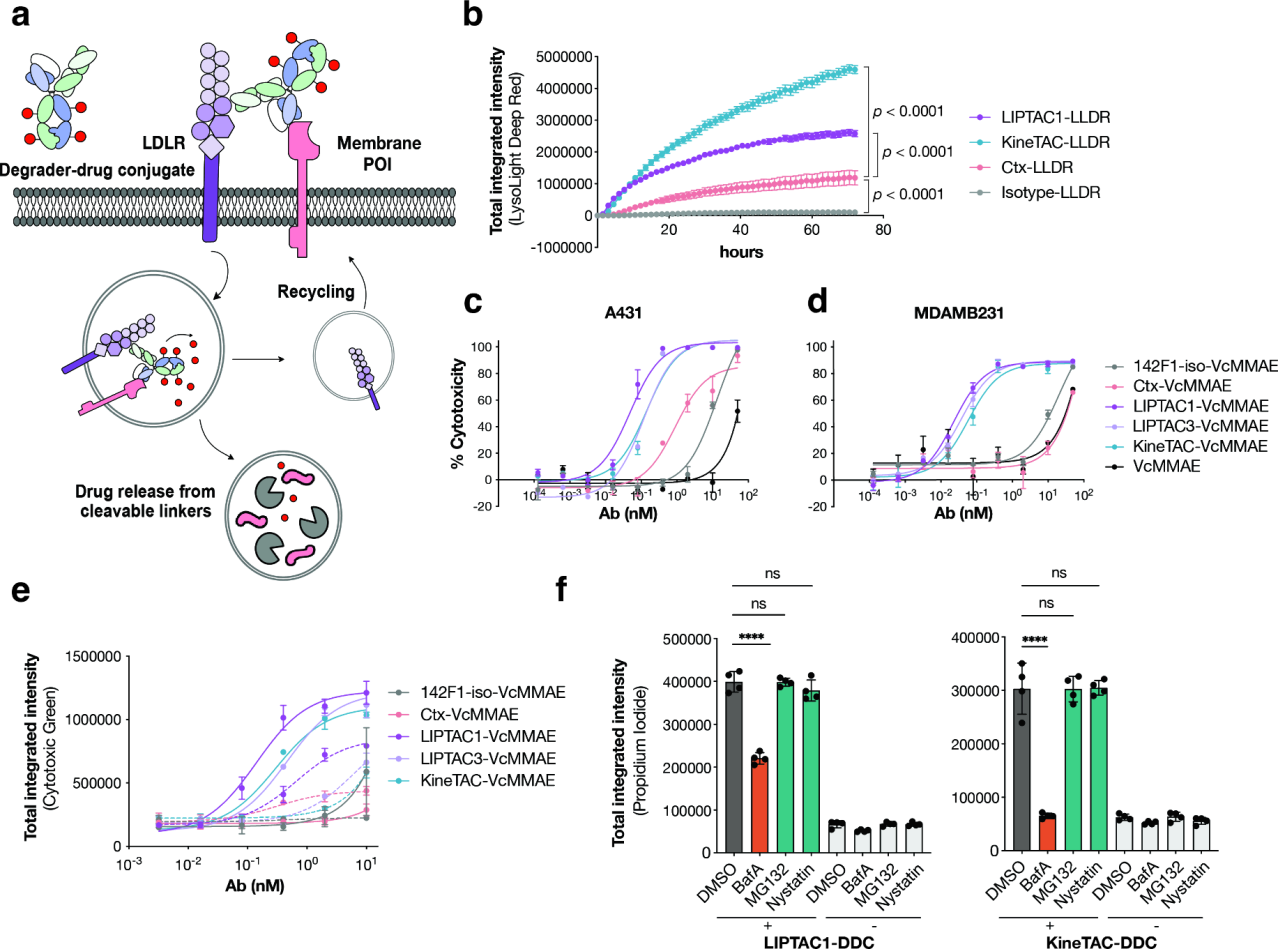
Development of degrader–drug conjugates (DDCs) for potent target cell killing. (a) Schematic illustration of LIPTAC-DDCs where the membrane POI is internalized by endocytosis and the cytotoxic payload is released from cleavable linkers in the lysosome. (b) A payload cleavage assay in EGFR-expressing A431 cells. Cells were treated with 25 nM of antibodies conjugated with lysolight deep red (LLDR) dyes. Images were captured every 2 h for 72 h on the Incucyte. Total integrated intensity was calculated by NIRCU × μm^2^/image. Error bars represented standard deviations for four biological replicates. Statistics were calculated by one-way ANOVA and Holm–Sidak multiple comparisons test. (c,d) Cytotoxicity of Ctx-ADC, monomeric 142F1-ADC, and Ctx-DDC either in LIPTAC or KineTAC formats on A431 and MDA-MB-231 cells, respectively. After 72 h incubation, cell viability was measured using the CellTiter-Glo Reagent. EC_50_ values were calculated using “One-Site Fit LogIC50” regression in GraphPad Prism 10.2. (e) Cytotoxicity of ADCs and corresponding DDCs on A431 cells in the presence and absence of the lysosomal inhibitor, BafA. Dead cells were labeled by cytotoxic green dye after 48 h of treatment. Dashed lines represented 50 nM BafA treatment together with antibodies. Total integrated intensity was calculated by GreenCU × μm^2^/image on the Incucyte. (f) Cytotoxicity of 0.8 nM DDCs on A431 cells in the presence of 50 nM BafA, 50 nM MG132, and 1 μM Nystatin, respectively. Cells treated with inhibitors alone showed no cytotoxicity at the concentrations used in DDC treatments. Dead cells were labeled by 1 μg/mL propidium iodide after 48 h of treatment. Total integrated intensity was calculated by ROCU × μm^2^/image on the Incucyte. Error bars represent standard deviations of four biological replicates. Statistics were calculated by unpaired two-tailed student *t* test. ****P < 0.0001. ns, not significant.

To evaluate the drug release efficiency of DDCs, we conjugated a cathepsin-dependent fluorescent probe, LysoLight Deep Red (LLDR), to monitor the lysosomal catabolism of internalized proteins.^53^ The antibody–probe conjugate was linked via a valine-citrulline (Vc) linker and remains nonfluorescent until cleavage by lysosomal cathepsins, which generates a bright fluorescent signal and serves as a surrogate for an ADC. We found that Ctx-DDC based conjugates, in either a KineTAC or LIPTAC form, showed much more efficient internalization and lysosomal trafficking compared to Ctx-IgG ADC (Figure 4b, Figure S7a), suggesting enhanced drug delivery efficiency for the DDC.

Next, we evaluated the toxicity of Ctx-DDC by conjugating it to VcMMAE as the cytotoxic payload. MMAE is an auristatin derivative that inhibits tubulin polymerization with a cleavable VC linker.^54^ To compare the cell killing potency of DDCs and Ctx ADC, we conjugated VcMMAE to monomeric 142F1, LIPTAC1, LIPTAC3 (bispecific format with 142F1 scFv and Ctx Fab), KineTAC, and Ctx IgG, respectively (Figure S7b). The average drug–antibody ratio (DAR) was 2.6, as estimated using 2,4-dinitrophenol (DNP)-PEG4 conjugation with the same method (Table S2). First, we confirmed that antibodies after drug conjugation retained binding to EGFR^+^ cells (Figure S7c). Next, we evaluated the potencies of the DDCs in the EGFR^high^ A431 cells. The Ctx ADC exhibited an IC_50_ of 0.9 nM, while the LIPTAC and KineTAC DDCs significantly enhanced the potency by 18-fold and 7-fold, respectively (Figure 4c). We then assessed the cytotoxicity in EGFR^medium^ MDA-MB-231 cells, which others have shown are relatively insensitive to Ctx-VcMMAE due to the low receptor expression.^55^ After 72 h of treatment, the Ctx ADC induced only moderate cytotoxicity, even at the highest ADC concentration (Figure 4d). The single arm anti-LDLR 142F1-VcMMAE also exhibited weak cell killing potency, suggesting that LIPTAC binding is primarily driven by the higher-affinity Ctx arm (Figure 4d, Figure S7c). Remarkably, all bispecific DDCs, including LIPTAC1-VcMMAE, LIPTAC3-VcMMAE, and KineTAC-VcMMAE, potently killed the target cells (Figure 4d), with IC_50_ values of 23 pM, 38 pM, and 59 pM, respectively.

We observed similar DDC-mediated killing potency on PANC-1 cells, which expressed similar levels of EGFR as MDA-MB-231 (Figure S7d). None of the antibodies without payload were significantly toxic to cells over 3 days of treatment (Figure S7e), suggesting that the cytotoxicity is mediated by internalization and release of the degrader conjugated MMAE. The cytotoxicity of ADC and DDCs was minimal on EGFR negative MCF7 cells and K562 cells, whereas free MMAE effectively killed these cells (Figure S7f,g). These results demonstrate that cytotoxicity is mediated by specific antibody-directed delivery rather than adventitious drug release and that the tumor-associated antigen arm dictates target cell recognition.

We also evaluated the lysosomal dependency of cell killing using an incucyte-based killing assay. Accumulation of cell death was still detectable when cells were treated with as low as 0.4 nM DDCs, while the cell death was not detectable with Ctx-ADC or 142F1-ADC (Figure 4e, Figure S7h). Bafilomycin A treatment abolished the cytotoxicity of both ADC and DDCs, further indicating the requirement of lysosomal release of the cytotoxic payload (Figure 4e). We further treated cells with chemical inhibitors of several endocytic pathways. Inhibitors of proteasomes (MG132) or caveolar-mediated endocytosis (Nystatin)^56,57^ did not significantly affect the cytotoxicity of the DDCs, highlighting receptor-mediated lysosomal delivery (Figure 4f). Together, these data suggest that DDCs improve lysosomal delivery of cytotoxic payloads and enhance the cytotoxic potency.

### Degrader–Drug Conjugates Improved Cytotoxicity on Cells with Moderate Antigen Expression Levels

To evaluate whether DDCs can enhance the drug delivery efficiency of these ADCs, we selected several clinically relevant ADC targets: B cell maturation antigen (BCMA, target of Belantamab^58,59^ ), cleaved CDCP1 (target of CL03 ^52^), and trophoblast cell surface antigen 2 (TROP2, target of Sacituzumab^60^). We engineered these therapeutically relevant antibodies into corresponding LIPTAC formats and conjugated them to VcMMAE at DARs similar to those of EGFR-targeting DDCs.

BCMA is highly expressed in malignant plasma cells and represents a validated target in multiple myeloma. Belantamab mafodotin (Blenrep) is a novel ADC composed of an anti-BCMA antibody conjugated to MMAF via a noncleavable linker.^60^ In RPMI-8226 cells, which express moderate levels of BCMA (normalized transcript per million (nTPM) = 171), Belantamab-LIPTAC demonstrated more efficient internalization than Belantamab (Figure 5a). Both ADCs and DDCs retained binding affinity comparable to that of their unconjugated counterparts (Figure S8a). In the luciferase-based viability assay, LIPTAC DDCs improved potency in RPMI-8226 cells by 4-fold, reducing the EC_50_ from 85 to 22 nM. The effect was not observed in MM1.S cells with higher BCMA expression (nTPM = 481) (Figure S8b). Notably, RPMI-8226 and MM1.S cells express comparable levels of LDLR (nTPM = 41 and 55, respectively). By annexin V and propidium iodide staining, LIPTAC DDCs significantly enhanced cell death by 18-fold in RPMI-8226 and 5-fold in MM1.S cells (Figure 5b, Figure S8c). This difference may be due to incomplete killing by DDCs in BCMA^+^ cells, where annexin V staining captures early apoptotic events. Overexpression of BCMA in RPMI-8226 cells rendered both LIPTAC DDC and Belantamab ADC comparably potent (Figure S8d,e), suggesting that LIPTAC-mediated trafficking offers the greatest advantage in cells with moderate antigen expression.

**Figure 5:**
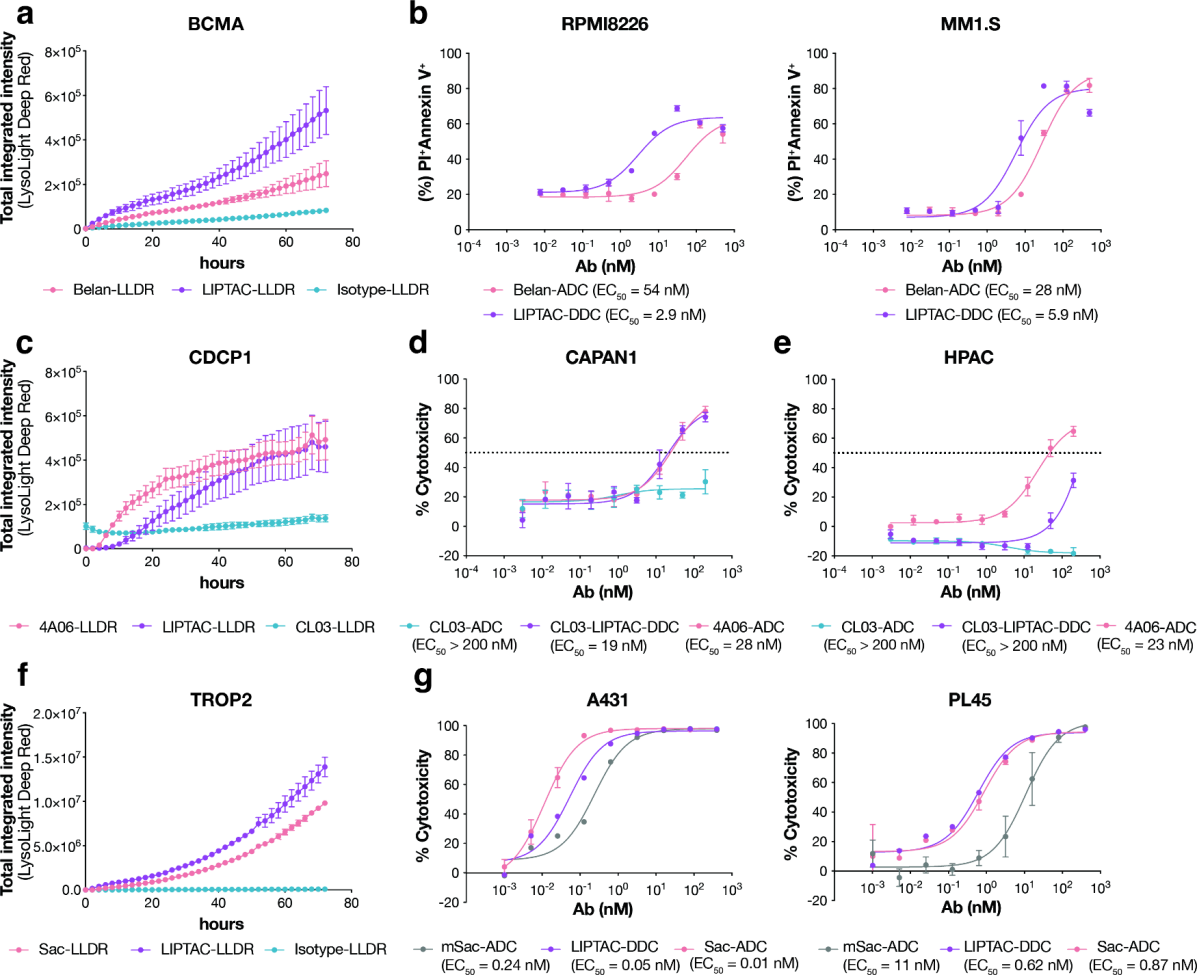
Comparison of clinically relevant ADCs and the DDC counterparts. (a) Antibody internalization assay in RPMI8226 cells, a multiple myeloma cell line, treated with 100 nM of LLDR dye-labeled antibodies. An anti-SARS-CoV-2 spike antibody CC12.1 ^74^ was used as the isotype control. Images were captured every 2 h for 72 h on the Incucyte. Each sample was tested in biological triplicate, and error bars represent the standard deviations. (b) Cytotoxicity of anti-BCMA Belantamab (Belan) ADC or the corresponding LIPTAC-DDC after 4 days incubation with RPMI8226 and MM1.S cells. To monitor cell death, cells were stained with APC-annexin V and propidium iodide (PI) and analyzed by flow cytometry. Each sample was tested in biological triplicate and error bars represent standard deviations. (c) Antibody internalization assay in CAPAN1 cells, a pancreatic cancer cell line, treated with 100 nM of LLDR dye-labeled antibodies. Images were captured every 2 h for 72 h on the Incucyte. Each sample was tested in biological triplicate and error bars represent the standard deviations. (d,e) Cytotoxicity of pan-CDCP1-targeting 4A06 ADC, cCDCP1-targeting CL03-ADC, or cCDCP1-targeting LIPTAC-DDC after 48 h incubation with (d) CAPAN1 cells or (e) HPAC cells. Cell viability was measured using the CellTiter-Glo reagent. Each sample was tested in biological triplicate and error bars represent standard deviations. (f) Antibody internalization assay in TROP2-expressing A431 cells treated with 100 nM of LLDR dye-labeled antibodies. Images were captured every 2 h for 72 h on the Incucyte. Each sample was tested in biological triplicate and error bars represent the standard deviations. (g) Cytotoxicity of monomeric Sacituzumab (mSac)-ADC, Sac-ADC, and LIPTAC-DDC after 3 days incubation with TROP2^high^ A431 or TROP2^medium^ PL45 cells. Each sample was tested in biological triplicate and error bars represent standard deviations. EC_50_ values were calculated using “One-Site Fit LogIC50” regression in GraphPad Prism 10.2.

We next examined CDCP1, a protein overexpressed in RAS-driven cancers. The cleavage-specific antibody CL03 has previously been shown to localize to tumors and exhibit antitumor activity with improved safety profiles,^52^ but it demonstrated limited internalization (Figure 5c) and inefficient degradation of cCDCP1 (Figure 3f). In contrast, CL03-LIPTAC internalized efficiently in pancreatic cancer CAPAN1 cells, comparable to the pan-CDCP1-targeting antibody 4A06 (Figure 5c). Moreover, the cCDCP1-directed DDC induced cytotoxicity in CAPAN1 cells to a level similar to that of the pan-CDCP1-targeting 4A06-ADC, whereas CL03-ADC showed no activity (Figure 5d). Importantly, neither the cCDCP1-targeting ADC nor DDC exhibited cytotoxicity in HPAC cells (Figure 5e), which lack proteolytically cleaved CDCP1,^52^ further underscoring the selectivity of this approach.

Then, we investigated TROP2, a clinically validated ADC target of sacituzumab govitecan,^59,61,62^ which carries an average DAR of 7.6. TROP2 is known to undergo rapid internalization upon antibody binding, with a reported internalization half-life (*t*_1/2_) of approximately 30 min.^68^ TROP2-LIPTAC showed a modest enhancement in internalization (Figure 5f). While the LIPTAC DDC did not further increase cytotoxicity in TROP2^high^ A431 (nTPM = 1085) or TROP2^medium^ PL45 (nTPM = 358) cells (Figure 5g), it demonstrated substantially greater potency compared with the monomeric sacituzumab ADC (Figure 5g). These findings suggest that avidity effects or receptor clustering^9,63^ may enhance lysosomal trafficking and payload delivery, supporting the potential of multispecific antibody designs with two antigen-binding arms and one recycling receptor arm to improve drug delivery against rapidly internalizing targets such as TROP2.

Overall, these results indicate that DDCs can provide added benefit in contexts where the parental ADC is limited, such as target-low settings, or when internalization is inefficient. When the ADC is in high abundance and a good recycler, the benefit of a DDC is clearly less. These findings underscore the importance of target selection and receptor biology in guiding the DDC design.

## Discussion

DDCs represent a new class of hybrid therapeutics that intentionally integrates the efficient lysosomal trafficking of eTPD with the cytotoxic payload delivery of ADCs. Our results show that DDCs outperform conventional ADCs in lysosomal trafficking and cytotoxic activity, underscoring the importance of intracellular routing as a key determinant of ADC efficacy. In head-to-head comparisons, EGFR-targeting Ctx-DDCs outperformed Ctx-based ADCs in terms of lysosomal trafficking and cytotoxicity. Tumor selectivity was conferred by the high-affinity Ctx arm,^64^ as LDLR-targeting alone exhibited weak activity. DDCs exhibited minimal toxicity to cells without EGFR expression, further highlighting that DDC specificity stems from the tumor antigen-binding domain.

Within this framework, the LIPTACs extend the scope of eTPD by exploiting LDLR’s exceptionally rapid and durable recycling capacity. LDLR can complete one endocytic cycle approximately every 12 min and recycle up to 150 times.^65^ LIPTACs enabled efficient and selective degradation of diverse membrane proteins and further demonstrated that the LDLR can function as a powerful trafficking hub to enhance payload delivery in the DDC format. We have not observed a hook effect, though this may depend on POI and LDLR expression levels and the very high concentrations typically required to reach the hook zone. More broadly, eTPD technologies such as LIPTAC and KineTAC complement ADCs by ensuring robust lysosomal delivery, thereby addressing a key bottleneck in ADC pharmacology.^66^ Our data suggest that LIPTAC is recycled after lysosomal delivery, while its payload is cleaved and released in the lysosome. Whether LIPTAC acts catalytically remains unclear. Lysosomal fluorescent signals appear within 4–6 h and plateau by 72 h, consistent with typical eTPD kinetics.^5^ These findings indicate that LDLR recycling and payload release occur on similar time scales, though precise rate comparisons will require future quantitative studies.

The therapeutic potency of ADCs depends on the antigen abundance, receptor internalization, and payload potency. Our results suggest that DDCs can provide a distinct advantage in settings where conventional ADCs are limited, such as receptors with low expression or poor internalization (e.g., BCMA on RPMI-8226 cells and cCDCP1 on CAPAN1 cells). This benefit was less pronounced for highly expressed or efficiently internalizing antigens, as seen with BCMA-overexpressing cells or TROP2-expressing cells, where the DDC format performed comparably to that of benchmark ADCs. Reduced avidity and lower binding affinities of bispecific DDCs likely also contribute to these differences, pointing toward future engineering opportunities.

The efficiency of target degradation depends on the balance of the affinities between the tumor-targeting and recycling arms of bispecific DDCs. To increase selectivity, it is critical that the tumor-binding affinity be high while the recycling affinity be low, ensuring that the DDC engages tumor antigens first rather than the recycling receptor. Future designs may incorporate multispecific formats, but the choice of recycling receptors must emphasize tumor-specific expression to minimize on-target, off-tumor activity.

The modularity of the DDC format suggests broader applications beyond the classical tumor antigens. It may expand druggability to unconventional, tumor-selective targets such as post-translational modifications^67,68^ and tumor-specific neo-antigens.^69^ Optimization of antigen-targeting arms and the use of heterobifunctional degraders could reduce DAR requirements, minimize off-target effects, and broaden the therapeutic window.

Finally, a major limitation of eTPD approaches is incomplete degradation,^70^ which may be insufficient to fully suppress oncogenic signaling. The DDC format overcomes this by delivering cytotoxic payloads directly to tumor cells independent of full protein clearance. This platform can be expanded to carry diverse therapeutic cargos including radionuclides, kinase inhibitors, or proteolysis-targeting chimeras (PROTACs). Together, the DDC modality represents a promising next-generation hybrid strategy for targeted cancer therapy.

## Materials and Methods

### Plasmid Construction

All the IgGs were constructed in a pcDNA3.4 vector that expresses the light chain and heavy chain, respectively, for mammalian expression. For generating bispecific antibody, the heavy chain variable regions or scFvs were cloned into zymework-A mutant Fc and zymework-B Fc sequences, respectively. Antigen was cloned into pFuse vector with IL-2 signal peptide followed by tobacco etch virus (TEV) protease, Fc, and Avitag sequences in the C-terminus. All of the Fabs were constructed in a dual-expression pBL347 vector that expresses the light chain and the heavy chain with the pelB and the stII signal peptides, respectively, for the periplasm expression.

### Cell Lines

HEK293T, HeLa, MDA-MB-231, PANC-1, A431, and HPAC cells were cultured in DMEM (ThermoFisher Scientific) with 10% fetal bovine serum (FBS) and 1% penicillin–streptomycin. MCF7 cells were cultured in DMEM with 10% FBS, 1% penicillin–streptomycin, 1% sodium pyruvate, and 1% nonessential amino acid. NCI-H1975, HCC1143, PL45, BT474, Raji, Ramos, RPMI8226, and MM1.S cells were cultured in RPMI-1640 (ThermoFisher Scientific) with 10% FBS and 1% penicillin–streptomycin. CAPAN-1 cells were cultured in IMDM with 10% FBS, 1% penicillin–streptomycin. HCC1143 LDLR KO cells were provided by Dr. James Olzmann and were supplemented with 100 μg/mL hygromycin (Sigma-Aldrich). of Expi293 cells were cultured in FreeStyle 293F medium (ThermoFisher Scientific, catalog no. A1435103).

### Phage Display

Phage selection was done as described previously.^71^ In brief, library E from the University of Chicago and the UCSF library were incubated with streptavidin-coated magnetic beads preconjugated with biotinylated Fc protein to remove nonspecific binders. Unbounded phage were then incubated with streptavidin-coated magnetic beads preconjugated with biotinylated cLDLR-TEV-Fc antigens. After 4 washes, antigen-bound phages were eluted from beads by incubating with 1 μM TEV protease for 20 min. In total, four rounds of selections were performed with decreasing concentrations of cLDLR-antigen (1000, 50, 20, and 10 nM). From round 3, the phage library was first enriched by protein A magnetic beads to deplete nondisplayed or truncated Fab phages before each round of the selection.

### Phage ELISA

First, 384-well Maxisorp plates were coated with Neutravidin (10 μg/mL) overnight at 4 °C and subsequently blocked with BSA (2% w/v) for 1 h at RT. Then 20 nM biotinylated cLDLR antigens were captured on the NeutrAvidin-coated wells for 30 min, followed by the addition of 1:5 diluted single-colony phage for 1 h. The secondary antibodies were either a horseradish peroxidase (HRP)-conjugated anti-M13 phage antibody (Sino Biological) for phage ELISA or an antihuman IgG antibody (Sigma-Aldrich) for recombinant protein ELISA. The ELISA plates were washed three times after each incubation, and antibody binding was detected by TMB substrate (VWR) and read at 450 nm.

### Protein Expression

IgGs and antigens were expressed in Expi293F cells on a 30 mL scale. In brief, 24 μg of DNA was added to 3 mL of OptiMEM, followed by 24 μL of the FectoPro transfection reagents. After 10 min of incubation, 27 mL of Expi293F cells at 3 million/mL was added and shaken at 37 °C. On the second day, 300 μL of 300 mM vaporic acid and 270 μL of 45% glucose were added to the cells. After 5 days of transfection, cells were harvested, spun down at 4000*g* for 20 min, and filtered by 0.45 μm steri-flip. Supernatants were then incubated with Sepharose A resin for 2 h, and proteins were then eluted by 0.1 M acetic acid and neutralized by Tris pH 11. Proteins were buffer changed 3 times in PBS in amicon tubes. Fabs were expressed in *Escherichia coli* C43 (DE3) Pro+ grown in an optimized TB autoinduction medium at 37 °C for 6 h, cooled to 30 °C for 18 h. Cells were harvested by centrifugation and lysed using B-PER lysis buffer. The lysate was incubated at 60 °C for 20 min and centrifuged to remove the inclusion body. The Fabs were purified by Sepharose A resin via affinity chromatography and buffer exchanged in PBS for further characterization. Purity and integrity of all proteins were assessed by SDS–PAGE.

### Recombinant Protein ELISA

First, 384-well Maxisorp plates were coated with Neutravidin (10 μg/mL) or 6x histag antibody (Invitrogen, catalog no. MA1-21315, 2 μg/mL) overnight at 4 °C and subsequently blocked with BSA (2% w/v) for 1 h at RT. Then 20 nM antigens were captured onto precoated wells for 1 h. Recombinant full-length LDLR, LRP2, LRP8, and VLDLR proteins were purchased from ACROBiosystems. For polyspecificity ELISAs, the autoantigens cardiolipin (Sigma, 50 μg/mL), insulin (Sigma, 1 μg/mL), lipopolysaccharide (LPS, InvivoGen, 10 μg/mL), and single-stranded DNA (ssDNA, Sigma, 1 μg/mL), were directly coated onto plates overnight 4 °C. After three washes, serially diluted Fabs were added to the plates and incubated for 1 h at RT. After three washes, 1:5000 diluted peroxidase–antihuman IgG (H+L) (Jackson ImmunoResearch) were added to the plates and incubated for 30 min. After three wash cycles, antibody binding was detected by TMB substrate (VWR), quenched by 1 M phosphoric acid, and read at 450 nm.

### Flow Cytometry

Cells were collected by centrifugation at 400*g* for 5 min. Pellets were washed once with PBS + 1% BSA. Cells were incubated with fluorophore-conjugated antibodies in PBS + 1% BSA for 15 min at RT or 30 min at 4 °C. Cells were washed three times and resuspended in cold PBS for flow analysis. Antibodies used included APC anti-LDLR (Invitrogen, catalog no. MA5-40994, 1:400), PE antihuman EGFR (Invitrogen, catalog no. MA5-28544, 1:400), Alexa fluor 647 goat antihuman IgG (H+L) (Invitrogen, catalog A-21445, 1:1000), APC annexin V (BioLegend, catalog no. 640920, 1:500), Alexa Fluor 647-conjugated protein A (Invitrogen, catalog no. P21462, 1:1000). Dead cell staining included propidium iodide (Biolegend, catalog no. 421301, 1:250), and a LIVE/DEAD fixable violet dead cell stain kit (Invitrogen, catalog no. L34964). Flow cytometry was performed using a CytoFLEX cytometer (Beckman Coulter, version 2.3.1.22) and CytoExpert software (version 2.3.1.22). Data were analyzed with FlowJo (v.10.8.0).

### Biolayer Interferometry

BLI experiments were performed at room temperature by using an Octet RED384 instrument (ForteBio), 20 nM biotinylated antigen-Fc was immobilized on an optically transparent SA biosensor (ForteBio). Different concentrations of monomeric Fab antibodies in kinetic buffer (PBS, 0.05% Tween-20, and 0.2% BSA) were used as the analyte in a 384-well microplate (Greiner Bio-One). Because the Fc format presents antigens bivalently on the sensor surface, affinities (*K*_D_s) were best fit with a 2:1 heterogeneous ligand model and calculated by a global fit analysis using the Octet RED384 Data Analysis HT software.

### Epitope Binning by BLI

Anti-LDLR antibodies were binned into epitope specificities using an Octet RED384 system. Then 20 nM biotinylated cLDLR-Fc antigens were captured using streptavidin biosensors (Fortebio). After antigen loading, a saturating concentration of antibodies (200 nM) was added for 10 min. Competing concentrations of antibodies (40 nM) were then added for 5 min to measure the binding in the presence of saturating antibodies. All incubation steps were performed in PBS/0.05% Tween-20/0.2% BSA. For PCSK9 epitope binning, all incubation steps and protein dilution were performed at acidic endosomal pH to increase PCSK9 binding affinity.^72^ Then 200 nM concentration of PCSK9 D374Y (AcroBiosystems) was added for 10 min after antigen loading. Then 40 nM PCSK9 D374Y, 142F1, or 142F6 was added for 5 min.

### LDL Uptake Assay

LDL uptake was measured by the Image-iT pHrodo Red Low Density Lipoprotein Uptake Kit (Thermo Scientific, catalog no. I34360) following manufacturer’s instructions. Briefly, HeLa cells were seeded at 5000 cells/well on a 96-well polystyrene tissue culture treated plate (Corning, catalog no. 3596). The next day, media was removed, and cells were serum starved for 12 h. Next, cells were pretreated with unlabeled LDL, heparin, and anti-LDLR Fabs for 30 min at 37 °C, respectively, followed by pHrodo red-labeled LDL treatment. Cells were then imaged by Incucyte (Sartorius) every 1 to 2 h. Internalization was calculated by total integrated intensity (ROCU × μm^2^/image) on the Incucyte software.

### Degradation Experiments

Cells were plated in 6- or 12-well plates and grown to ∼70% confluency before treatment. On the next day, cell culture medium was aspirated, and various concentrations of antibodies in 1 mL of culture medium were then added to each well. Cells were incubated for 24 h at 37 °C prior to flow cytometry or Western blotting experiments.

### Western Blotting

Cells were lifted with PBS + 0.05% EDTA, transferred to Eppendorf tubes, spun down at 500 g for 4 min, and washed twice with PBS. Cells were lysed with 1× RIPA lysis buffer (EMD Millipore) with cOmplete mini protease inhibitor cocktail (Sigma-Aldrich) at 4 °C for 20 min. Lysates were centrifuged at 20,000*g* for 10 min at 4 °C to remove debris. Soluble protein concentrations were quantified by Rapid Gold BCA Protein Assay Kit (Pierce). Lysates were mixed with 4× Nupage LDS Sample Buffer (Invitrogen) and 2-mercaptoethanol, and then run on NuPAGE 4–12% Bis Tris Protein Gels (Thermo Fisher Scientific). Proteins were transferred to polyvinylidene difluoride membranes using the iBlot2 Western Blotting Transfer System (Thermo Scientific). Membranes were blocked with TBS + 5% BSA + 0.5% Tween for 1 h, and stained with primary antibodies overnight. After three washes, membranes were stained with secondary antibodies for 1 h at RT. After three washes, membranes were imaged with a LICOR imager or the ChemiDoc MP imaging system (BioRad). Antibodies used included rabbit anti-human EGFR (Cell Signaling Technology, catalog no. 4267S, 1:1000), rabbit anti-human PD-L1 (Cell Signaling Technology, catalog no. 13684S, 1:1000), rabbit anti-human CXCR4 (Cell Signaling Technology, catalog no. 64837S, 1:1000), rabbit anti-human ERBB2 (Cell Signaling Technology, catalog no. 4290S, 1:1000), rabbit anti-human CDCP1 (Cell Signaling Technology, catalog no. 13794S, 1:1000), mouse anti-human α-tubulin (Cell Signaling Technology, 3873S, 1:3000), goat anti-human LDLR (R&D Systems, catalog no. AF2148, 1:1000), IRDye 800CW goat anti-rabbit IgG (LI-COR Biosciences, catalog no. 926-32211), IRDye 680RD goat anti-mouse IgG (LI-COR Biosciences, catalog no. 926-68070, 1:5000), IRDye 800CW donkey anti-goat IgG (LI-COR Biosciences, catalog no. 926-32214, 1:5000), and peroxidase goat anti-rabbit IgG (H+L) (Jackson ImmunoResearch, catalog no. 111-035-144, 1:5000).

### Confocal Microscopy

HeLa cells were plated on the chambered coverslip (Ibidi, 8-well uncoated) and incubated for 24 h at 37 °C. Cells were then treated with 50 nM bispecific or control antibodies in complete growth medium. After 24 h of incubation at 37 °C, medium was aspirated, and cells were washed with PBS. Cells on the coverslips were fixed with paraformaldehyde (PFA) for 15 min at RT, then permeabilized with 0.1% Triton-X in PBS for 10 min at RT. After three washes with PBS, the samples were stained with anti-LAMP1 rabbit antibody (Cell Signaling Technology, cat. no. 9091T), anti-EGFR mouse antibody (Thermo Scientific, catalog no. MA5-13070), and DAPI (Cell Signaling Technologies). Goat antirabbit IgG 488 (Invitrogen, catalog no. A-11008) and goat antimouse IgG 647 (Invitrogen, catalog no. A-21240) were stained for visualization. Samples were imaged using a Nikon Ti Microscope with a Yokogawa CSU-22 spinning disk confocal and a 60× objective lens; 405-, 488-, and 647 nm lasers were used to image DAPI, LAMP1 and EGFR, respectively. Images were deconvoluted and processed by using NIS-Element (v5.21.03) and Fiji software (v2.1.0) packages.

### Cell Culture/Stable Isotope Labeling Using Amino Acids in Cell Culture (SILAC) Labeling

MDA-MB-231 cells were grown in DMEM for SILAC (Thermo Fisher) with 10% dialyzed FBS (Gemini). Medium was also supplemented with either light L-[^12^C_6_,^14^N_2_]-lysine/l-[^12^C_6_,^14^N_4_]-arginine (Sigma) or heavy L-[^13^C_6_,^15^N_2_]-lysine/L-[^13^C_6_,^15^N_4_]-arginine (Cambridge Isotope Laboratories). Cells were maintained in SILAC medium for five passages to ensure complete isotopic labeling. Heavy-labeled cells were treated with PBS control, and light-labeled cells were treated with 50 nM bispecific LIPTAC for 48 h before cells were collected. Cells were then used to prepare surface-proteome enrichment.

### Mass Spectrometry

For proteomic analysis, cells were processed following established cell surface capture methods.^38^ Approximately 2 million SILAC-labeled cells were first washed in PBS (pH 6.5) before the glycoproteins were mildly oxidized with 1.6 mM sodium periodate (Sigma) in PBS (pH 6.5) for 20 min at 4 °C. Cells were then biotinylated via the oxidized vicinal diols with 1 mM biocytin hydrazide (Biotium) in the presence of 10 mM aniline (Sigma) in PBS (pH 6.5) for 90 min at 4 °C. Cell pellets were lysed with a 2× dilution of commercial RIPA buffer (Millipore) supplemented with 1× protease inhibitor cocktail (Sigma) and 2 mM EDTA (Sigma) for 10 min at 4 °C. Cells were further disrupted with probe sonication (20% amplitude, 5 min, 4 °C), followed by cell debris removal (20,000*g*, 10 min, 4 °C), and the clarified cell lysates were then incubated with 50 μL of high-capacity NeutrAvidin-coated agarose beads (Thermo) in Poly-Prep chromatography columns (Bio-Rad) for 2 h at 4 °C to isolate biotinylated glycoproteins. To enrich for biotinylated proteins, the resin was washed sequentially with 5 mL of 1× RIPA (Millipore) plus 1 mM EDTA, 5 mL of high-salt PBS (20 mM phosphate (pH 7.4) with 1 M NaCl (Sigma)) and 5 mL of denaturing urea buffer (50 mM ammonium bicarbonate and 2 M urea). Proteins on the beads were next reduced, carbidomethylated, digested, and desalted using the Preomics iST mass spectrometry sample preparation kit (Preomics) per the manufacturer’s recommendations. After desalting, samples were dried, resuspended in 0.1% formic acid, and quantified using the Pierce peptide quantification kit (Thermo Scientific) before liquid chromatography–tandem mass spectrometry analysis.

Liquid chromatography–tandem mass spectrometry was performed by using a Bruker NanoElute chromatography system coupled to a Bruker timsTOF Pro mass spectrometer. Peptides were separated using a prepacked IonOpticks Aurora (25 cm × 75 μm) C18 reversed-phase column (1.6-μm pore size, Thermo) fitted with a CaptiveSpray emitter for the timsTOF Pro CaptiveSpray source. For all samples, 200 ng of resuspended peptides was injected and separated using a linear gradient of 2–23% solvent B (solvent A: 0.1% formic acid and 2% acetonitrile; solvent B: acetonitrile with 0.1% formic acid) over 90 min at 400 μL min 1 with a final ramp to 34% B over 10 min. Separations were performed at a column temperature of 50 °C. Data-dependent acquisition was performed using a timsTOF PASEF tandem mass spectrometry method (TIMS mobility scan range of 0.70–1.50 V•s cm^2^, mass scan range of 100–1700 *m*/*z*, ramp time of 100 ms, 10 PASEF scans per 1.17 s, active exclusion of 24 s, charge range of 0–5 and minimum MS1 intensity of 500). The normalized collision energy was set at 20.

### Mass Spectrometry Data Analysis

LC-MS-MS data was analyzed using PEAKS online Xpro 1.6 (Bioinformatics Solutions Inc.; Ontario, Canada). Spectral searches were performed in PEAKS Q (de novo assisted Quantification) mode. The precursor mass error tolerance was set to 20 ppm and the fragment mass error tolerance was set to 0.5. Peptides containing 6 and 45 amino acids in length were then searched in a semispecific trypsin/LysC digest mode against a proteome file that contains human cell surface proteins. Carbamidomethylation (+57.0214 Da) on cysteines was a set static modification; methionine oxidation (+15.994) and the isotopic labels (13C(6)­15N(2); 13C(6)­15N(4)) were set variable modifications. Quantified peptides were matched between three experimental replicates, and peptide enrichments were normalized based on the total ion chromatograph (TIC). SILAC-labeled protein ratios were further analyzed if the proteins were identified by more than one peptide and were present in at least two experimental replicates. A *p*-value of 0.05 and 2-fold protein ratio differences were set as cutoffs to determine if protein abundance differences were significant between vehicle treated cells and LIPTAC-treated cells. Proteomic data are available on PRIDE with PXD064642 accession code.

### Lysolight Deep Red Assay

Antibodies were labeled using the LysoLight Antibody Labeling Kits (Invitrogen, cat. no. L36003) following manufacturer’s instructions. Briefly, antibodies were labeled with LLDR with a molar ratio of 1:6 in the presence of 100 mM sodium bicarbonate (pH 8.4) for 2 h at RT. Antibodies were then purified with 7k Zeba dye and biotin removal columns (Thermo Scientific, catalog no. A44297). Cells were seeded at 5000/well on a 96-well polystyrene tissue culture treated plate (Corning, catalog no. 3596). The next day, media was removed, treated with LLDR-labeled antibodies, and then imaged on the Incucyte every 2 h for 72 h.

### *In Vitro* ADC Assays

IgGs were labeled with NHS ester-PEG4-ValCit-PAB-MMAE (BroadPharm, catalog no. BP-25503) with a 1:6 or 1:10 molar ratio at RT for 2 h with 100 mM sodium bicarbonate. Antibodies were then desalted using Pierce Zeba desalt spin columns (Thermo Scientific). For adherent cells, 5000 cells/well were seeded on a 96-well polylysine-coated white plate (Corning, catalog no. 3917). The next day, media was aspirated, and MMAE-labeled IgGs were added and incubated for 72 h in cell culture medium at 37 °C. For suspension cells, 6000 cells/well were incubated with ADCs or DDCs for 96 h at 37 °C. Viability was measured using CellTiter-Glo Reagent (Promega). For the incucyte-based killing assay, the cells were treated with ADCs or DDCs in cell culture medium at 37 °C, cytotoxic green dye (Sartorius, catalog no. 4633, 1:10000) or propidium Iodide (Biolegend, catalog no. 42130, 2 μg/mL). For compound inhibitor assay, 0.8 nM DDCs were incubated with 1 μg/mL propidium iodide with or without 50 nM Bafilomycin A (Santa Cruz Biotechnology, catalog no. sc-201550A), 50 nM MG132 (Selleck Chemicals, cat. no. S2619), or 1 μM Nystatin (MedChem Express, cat. no. HY-17409), respectively.

### Characterization of DAR

IgGs were side-by-side labeled with DNP-PEG4-NHS ester (MedChem Express, cat. no. HY-140614) with the same molar ratio as the MMAE conjugation and incubated at RT for 2 h with 100 mM sodium bicarbonate. Antibodies were then desalted using the Pierce Zeba desalt spin columns (Thermo Scientific). The absorbance of conjugated antibodies at 280 and 360 nm was measured by UV–vis spectrophotometer. The correction factor (CF) was determined by measuring A280 and A360 of the pure 100 μM DNP-PEG4-NHS ester solution.

$$\mathrm{DAR}=\frac{A360}{\varepsilon \mathrm{DNP}}/\left(\frac{A280-\left(A360\times \mathrm{CF}\right)}{\varepsilon \mathrm{protein}}\right)$$

## Supplementary Material



## Notes

Phage display screening for LDLR-specific antibodies; cross-reactivity and polyspecificity of cLDLR Fabs; Epitope binning of cLDLR Fabs; LIPTAC-mediated EGFR degradation in different formats and on multiple cell lines; surface proteins on MDA-MB-231 cells having differential abundance with or without LIPTAC1 treatment; epitope binning of PCSK9 and anti-LDLR antibodies; payload delivery and cytotoxicity of EGFR-targeting DDCs in comparison with ADCs; Cytotoxicity of BCMA-targeting ADC and DDC; complementarity-determining region (CDR) sequences of LDLR antibodies; characterization of relative DAR; (PDF)
